# The Crosstalk of Apoptotic and Non-Apoptotic Signaling in CD95 System

**DOI:** 10.3390/cells13211814

**Published:** 2024-11-03

**Authors:** Kamil Seyrek, Johannes Espe, Elisabeth Reiss, Inna N. Lavrik

**Affiliations:** Translational Inflammation Research, Medical Faculty, Otto von Guericke University Magdeburg, 39106 Magdeburg, Germany; kamil.seyrek@med.ovgu.de (K.S.); johannes.espe@med.ovgu.de (J.E.); elisabeth.reiss@med.ovgu.de (E.R.)

**Keywords:** CD95, caspase, extrinsic apoptosis, non-apoptotic signaling, DISC

## Abstract

The mechanisms of CD95 (Fas/APO-1)-mediated extrinsic apoptotic pathway in cancer cells have been extensively studied. The majority of human cells express CD95, but not all these cells can induce extrinsic apoptosis. Accumulating evidence has shown that CD95 is a multifunctional protein, and its stimulation can also elicit non-apoptotic or even survival signals. It has become clear that under certain cellular contexts, due to the various checkpoints, CD95 activation can trigger both apoptotic and non-apoptotic signals. The crosstalk of death and survival signals may occur at different levels of signal transduction. The strength of the CD95 stimulation, initial levels of anti-apoptotic proteins, and posttranslational modifications of the core DISC components have been proposed to be the most important factors in the life/death decisions at CD95. Successful therapeutic targeting of CD95 signaling pathways will require a better understanding of the crosstalk between CD95-induced apoptotic and cell survival pathways. In this review, in order to gain a systematic understanding of the crosstalk between CD95-mediated apoptosis and non-apoptotic signaling, we will discuss these issues in a step-by-step way.

## 1. Introduction

CD95 (Fas/APO-1) is a type I transmembrane glycoprotein belonging to the death receptor (DR) family, which is a subfamily of the tumor necrosis factor receptor (TNFR) superfamily [1,2,3,4,5,6,7,8].

Similar to all members of the DR family, CD95 can induce both apoptotic and non-apoptotic signaling [9]. Human mature CD95 consists of 319 amino acids, with the molecular mass ranging from 45 kDa to 54 kDa due to post-translational glycosylation [10,11,12,13,14]. The N-terminal extracellular region of CD95 contains three cysteine-rich domains (CRDs) [5,14]. CRDs (CRD1, CRD2, and CRD3) are indispensable for CD95 signaling pathways. In fact, CRD2 and partly CRD3 are crucial for CD95 ligand (CD95L) recognition and binding, which is a key step in the initiation of both apoptotic and non-apoptotic signals [15,16]. Moreover, a domain (amino acid residues 59 to 82) residing within the N-terminal region of CRD1, termed the pre-ligand assembly domain (PLAD), was shown to prevent spontaneous apoptosis by influencing CD95 conformation [15,16,17,18]. The transmembrane domain (TM) of CD95 comprises 17 amino acids followed by the cytoplasmic portion, which encompasses an 80 amino-acid stretch designated as the death domain (DD) [5,19,20,21,22,23]. CD95 DD consists of six anti-parallel α-helices, which are involved in homotypic interactions with the other DD-containing proteins [21,24]. Involvement of the intracellular domain of CD95 in CD95-mediated apoptotic and non-apoptotic signaling has been clearly established [3,22,25,26,27].

The CD95-mediated apoptotic pathway is one of the best-studied death receptor (DR) signaling pathways [28]. Upon activation with its cognate ligand or with agonistic antibodies, CD95 DD interacts with DD of Fas-associated protein with death domain (FADD) to initiate DR signaling via the formation of the death-inducing signaling complex (DISC) [3,29,30]. FADD in turn recruits procaspase-8 by serving as a hub to activate this apical caspase, the activation of which leads to apoptotic cell death [31,32]. Specifically, in the process of binding to FADD, procaspase-8 forms so-called Death Effector Domain (DED) filaments that serve as a platform for dimerization and subsequent activation of procaspase-8 [33,34,35,36]. The majority of human cells were shown to express CD95, but some cell types are sensitive to extrinsic apoptosis while others are partially or completely resistant, suggesting the existence of additional molecular mechanisms controlling the suppression of apoptosis [37,38,39]. In line with this statement, cellular FLICE inhibitory proteins (c-FLIPs), major suppressors of extrinsic apoptosis, were also shown to be recruited to the DISC [29,40,41,42]. One long and two short c-FLIP isoforms, named Long (L), Short (S), and Raji (R) (i.e., c-FLIP_L_, c-FLIP_S_, and c-FLIP_R_), are the main proteins that regulate caspase-8 activity [30,32,33,43,44]. Short isoforms prevent procaspase-8 activation by interfering with the formation of procaspase-8 homodimers due to the generation of inactive heterodimers [32,35,45]. The long isoform, c-FLIP_L_, depending on its expression level and ratio to procaspase-8, can act both in a pro- as well as in an anti-apoptotic manner [43,46,47,48,49]. Specifically, upon high expression levels, c-FLIP_L_ inhibits apoptosis by preventing procaspase-8 homodimerization at the DISC. In contrast, upon intermediate levels of expression, c-FLIP_L_ enhances the activation of caspase-8 via the generation of procaspase-8/c-FLIP_L_ heterodimers [46,50]. Specifically, c-FLIP_L_ stabilizes the active center of procaspase-8 in procaspase-8/c-FLIP_L_ heterodimers and thereby promotes the catalytic activity of procaspase-8 [46,47,51,52,53,54,55,56,57,58,59].

Among all members of the DR family, CD95 has the highest similarity in signaling to the signal transduction of TRAIL-Rs. Similar to CD95, TRAIL-Rs also form the DISC and DED filaments, with the same core components [60,61]. In a similar fashion, caspase-8 is activated at the TRAIL-R DISC and non-apoptotic signaling is also induced upon TRAIL stimulation [34]. The signaling of TNF-R1 has a different route and, in fact, TNFα stimulation leads to the preferential activation of NF-κB, which is based on the different composition of the TNF-R1 receptor complex [62].

Since CD95 has long been viewed as the DR prototype, the overwhelming majority of studies exploring CD95 signaling have focused on CD95-mediated apoptosis. This cell death-biased view has overshadowed the cell death-independent functions of CD95 [63]. However, depending on the cellular context and/or on the initial concentration of cognate ligand, CD95 stimulation may also activate prosurvival pathways. Indeed, CD95 is reported to activate nuclear factor-κB (NF-κB) and mitogen-activated protein kinase (MAPK) pathways, the activation of which may lead to inflammation, proliferation, differentiation, regeneration, angiogenesis, and even tumor progression [23,64,65,66,67,68,69,70,71]. However, the molecular mechanisms by which the receptor switches from an apoptotic function to non-apoptotic signaling have been not clarified yet. The success of therapeutically targeting CD95 requires a better comprehension of the factors determining the balance of cell death and death-independent activities of CD95 signaling, which will be discussed below.

## 2. General Aspects of CD95-Mediated Apoptotic Signaling Pathway

### 2.1. Binding of CD95L to CD95 Leads to Oligomerization of Receptor

CD95 engagement by its cognate ligand, CD95L, initiates extrinsic apoptotic and non-apoptotic signaling [15,16]. In fact, the TNF Homology Domain (THD) of CD95L interacts with CRD2 and part of the CRD3 of CD95, followed by the homotrimerization of CD95 through homotypic CRD1 interactions [16,17,72,73,74,75,76,77]. Along with CRDs within the extracellular region, the proline-containing motif in the TM domain of CD95 was also shown to be involved in CD95 trimerization [78]. In support of this, mutations in the TM domain were shown to attenuate CD95L-induced apoptosis by destabilizing CD95 multimers [78]. In addition, the pace of receptor homotrimerization has been reported to rely on the amount of CD95L interacting with CD95 [15,16,17]. Previous studies have established that at the plasma membrane, CD95 monomers can spontaneously self-assemble into homodimers or homotrimers [5,15,16,17,24,79,80]. This pre-oligomerization of CD95 in unstimulated cells is crucial to form larger signaling platforms to initiate efficient apoptosis [16,81]. Importantly, spontaneous receptor oligomerization at the cell membrane is largely dependent on PLAD; however, the existence of other domains in CD95 exerting complementary roles for receptor oligomerization cannot be ruled out [5,16,17]. The possibility that the expression level of the receptor itself may be critical in spontaneous CD95 oligomerization also cannot be excluded.

The strength of CD95L stimulation also plays a crucial role in triggering the type of signaling. CD95L was shown to induce both apoptotic and non-apoptotic signaling such as NF-κB or MAPK activation. Upon triggering intermediate or high concentrations of CD95L, this stimulation typically leads to the demolition of the cell in case the apoptotic machinery is present [13,54,69]. The so-called low or threshold-level stimulations also lead to the induction of both apoptotic and non-apoptotic pathways [65,70,82]. However, the threshold level of CD95 stimulation might cause no cell death, leading to the survival of the cells. Furthermore, it was shown that threshold-level CD95 stimulation might result in bistability behavior, e.g., different outcomes for the cell fate within the cellular population with some cells dying and some surviving [70].

Importantly, the form of CD95L, e.g., membrane-bound versus soluble, and likely the resulting degree of CD95 oligomerization were shown to play an important role in the transduction of apoptotic versus non-apoptotic signals [9,76]. In particular, it was shown that soluble CD95L induces a non-apoptotic signal, while membrane-bound one induces cell death [83]. It might be speculated that the highly oligomerized CD95 provides a more stable platform for the assembly of DED filaments, which is, in turn, essential for effective apoptosis induction. The other hypothesis might be that CD95 with a low degree of oligomerization has a different conformation of the intracellular DD stretch, which leads to the preferential recruitment of proteins inducing a non-apoptotic signal. Indeed, the formation of a core protein complex at CD95 DD termed the motility-inducing complex (MISC) has been reported. MISC enables the recruitment of PI3K and the induction of non-apoptotic signaling as discussed below [84,85]. Furthermore, it was shown that not only does the degree of CD95 oligomerization play a role but also the density of cells expressing CD95. The latter in turn regulates the threshold of CD95 apoptotic versus non-apoptotic signaling [86].

### 2.2. Assembly of Death-Inducing Signaling Complex (DISC) and Death Effector Domain (DED) Filaments

**Figure 1:**
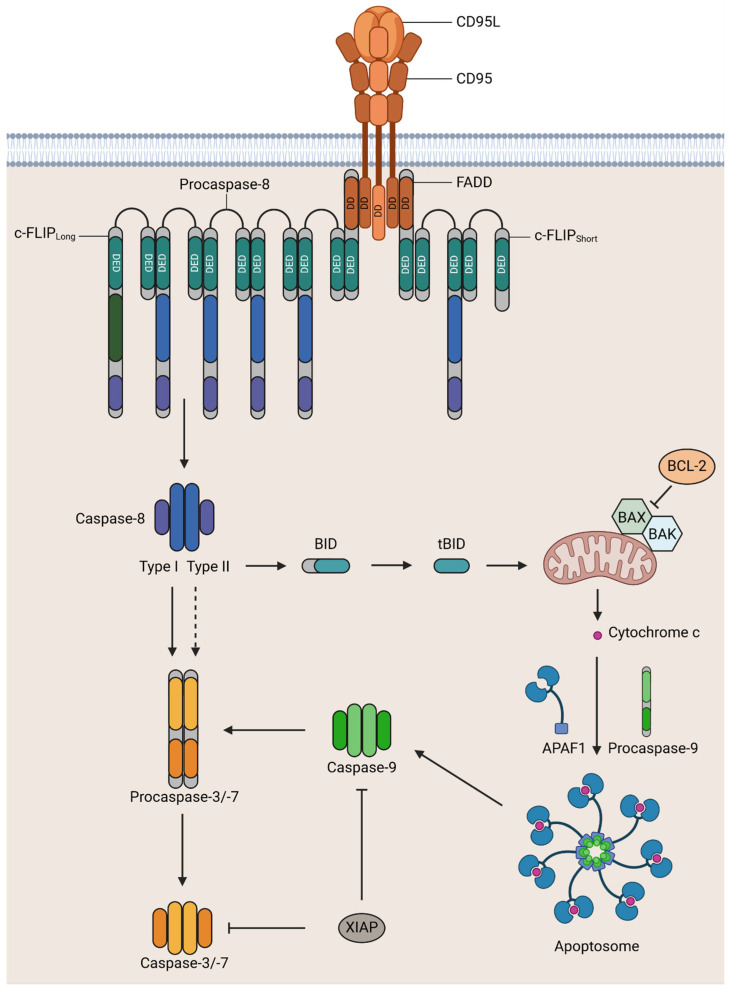
Scheme of CD95 pathway. The CD95 signaling pathway is of crucial importance for the homeostasis of multicellular organisms. Binding of CD95L to the CD95 initiates the signaling pathway and leads to assembly of the death-inducing signaling complex (DISC). Ligand binding results in a conformational change of the intracellular death domain (DD), which leads to the recruitment of FADD via its DD. FADD comprises a death effector domain (DED) through which it recruits other DED-containing DISC core proteins that form the DED filaments. These DED proteins include procaspase-8 and the c-FLIP isoforms. While c-FLIP_Short_ acts exclusively antiapoptotic, c-FLIP_Long_ can act both proapoptotic and antiapoptotic. At the DISC, procaspase-8 can be activated both as a homodimer and as a procaspase-8/c-FLIP_Long_ heterodimer and is cleaved to the heterotetrameric caspase-8. In type I cells, caspase-8 activation is sufficient to activate the effector caspases procaspase-3 and -7 and thus trigger apoptosis. In type II cells, amplification of the signal is necessary. In this case, caspase-8 cleaves BID to tBID, which in turn leads to permeabilization of the mitochondrial membrane and the release of cytochrome c. Cytochrome c, together with cytosolic APAF1 and procaspase-9, forms the apoptosome, which serves as a platform for caspase-9 activation. Caspase-9 activates the effector caspases and triggers apoptosis in type II cells. XIAP is an inhibitor of caspase-9 and the effector caspases. Through various stress signals, BAX and BAK are also able to permeabilize the mitochondrial membrane and trigger apoptosis in a CD95-independent manner. BCL-2 can inhibit BAX/BAK-mediated permeabilization. Figure created using BioRender.com, accessed on 26 September 2024.

Extensive work concerning the interaction between CD95 and its ligand revealed that the binding of CD95L triggers a reorganization of CD95 multimers and conformational changes in CD95 DD [26,41]. This structural new arrangement allows FADD DD binding to the CD95 DD [29,41,79,87,88]. In parallel, CD95L-induced conformational modification of DD drives the oligomerization of CD95 by bringing receptors in close proximity [41]. Hence, CD95L promotes DR aggregation and the recruitment of FADD onto the DD of CD95 through homotypic interactions followed by the self-association of FADD [89,90]. FADD self-association is crucial to form a stable CD95-FADD interaction, which drives DISC formation within seconds by attracting procaspase-8a/b, -10a/d, or c-FLIP_L/S/R_ to the CD95 [29,40,41,91,92,93] (Figure 1). It is well-documented that CD95-FADD complex-bound procaspase-8 has the potential to recruit additional procaspase-8 molecules, giving rise to the formation of an activation platform for procaspase-8 [33,34,78]. In fact, several procaspase-8 molecules interact with each other via tandem DEDs to form an activation platform, DED filaments, the generation of which promotes proximity-induced dimerization and subsequent autocatalytic cleavage of procaspase-8 [32,33,34,94]. DED filaments are composed of three linear DED chains, each of which is formed via interactions of the DEDs of procaspase-8 (Figure 1). Recent structural analysis has demonstrated that at the initial stage of assembly, trimerized FADD molecules bind triple procaspase-8 proteins via their DEDs, which forms the core of this complex, followed by the recruitment of further DED proteins and the elongation of three linear DED chains. In the same study, it was elucidated that at the later stages of DED filament assembly, proapoptotic procaspase-8 homodimers are formed at the first or third linear chain between subsequent procaspase-8 molecules [35]. Interestingly, the second chain seems to not be directly involved in the formation of proapoptotic dimers. During the elongation of this activation platform, along with procaspase-8 and FADD, other DED proteins including c-FLIP_L/S/R_ could also be incorporated into these DED filaments [30,33,94]. It was recently shown that the short isoforms of c-FLIP block the elongation of DED filaments by incorporation at the end of the DED chain [36]. In a more recent publication, it was shown that these inhibitory effects of c-FLIP are also linked to targeting the FADD–Procaspase-8 DED interaction within the intricate structure of the DED filament [35]. It is important to know that the catalytic activity of procaspase-8 in homodimers is limited to procaspase-8 itself [48,55,95,96]. Autocatalytic cleavage of procaspase-8a/b (p55/p53) occurs in a two-step process [97]. The first cleavage step produces the two subunits p43/p41 and p12. In the second cleavage step, prodomains p26/p24 and active enzyme subunits (i.e., p18 and p10) are generated [65,98]. Several studies have revealed that other cleavage products such as p30 and CAP3 (p27, quickly converted to p26) are also generated from procaspase-8a/b (p55/p53), indicating that the processing of procaspase-8 is more complicated than initially supposed [33,79,95,99,100,101,102]. Eventually, active p10 and p18 subunits are released into the cytoplasm to form mature caspase-8 heterotetramer (p10_2_-p18_2_), followed by the activation of downstream effector caspases-3 and -7 [34,95,102,103]. This series of events triggers the cleavage of several cellular proteins, such as fodrin, protein kinase C (PKC), gelsolin, poly (ADP-ribose) polymerase (PARP), inhibitor of caspase-activated DNase (ICAD), actin, and nuclear lamins followed by the demolition of the cells [11,32,33,104,105,106,107,108,109].

### 2.3. The Role of c-FLIP Proteins in CD95 Signaling

Structural studies have revealed that c-FLIP_L_ contains an activation domain in its C-terminus to initiate procaspase-8 activation [47,110]. Remarkably, the formation of the procaspase-8/c-FLIP_L_ heterodimers leads to more efficient activation of procaspase-8 than within the homodimers of procaspase-8 because the C-terminal part of c-FLIP_L_ has greater stabilization effects on the procaspase-8 C-terminal part than the procaspase-8 has for itself [53]. Further, procaspase-8 is cleaved more rapidly in heterodimers than that of caspase-8 proenzyme homodimers [46,69]. Additionally, upon activation, procaspase-8 cleaves c-FLIP_L_ in heterodimers, indicating that c-FLIP_L_ acts as an initiator of caspase-8 activation and its initial substrate. Accordingly, the loss of c-FLIP_L_ in mouse embryonic fibroblasts resulted in a decrease in procaspase-8 activation [110]. Some studies reported that the substrate specificity and catalytic activity of caspase-8 proenzyme in heterodimers are indistinguishable from those of procaspase-8 homodimers, but other studies have indicated that procaspase-8/c-FLIP_L_ heterodimers may have specificity toward a different set of substrates than that of homodimers [53,57,111]. For example, the procaspase-8/c-FLIP_L_ heterodimer but not the homodimer was shown to prevent receptor-interacting serine/threonine-protein kinase 1/3 (RIPK1/RIPK3)-dependent necrosis [59,112]. Moreover, uncleavable caspase-8 was shown to be activated by dimerization with c-FLIP_L_ [57]. Further, the p22-FLIP fragment is generated from heterodimers of procaspase-8 with c-FLIP_L_ by cleaving c-FLIP_L_ at D196 [13,94,110,113,114,115,116]. It is also important to note that procaspase-8 can also generate p22-FLIP from c-FLIP_S/R_ [113]. Hence, it can be speculated that the caspase-8 proenzyme in homodimers may have more distinct activities than those of those in the c-FLIP_L_-procaspase-8 complex [59].

Apart from its role in procaspase-8/c-FLIP_L_ heterodimers, c-FLIP_L_ has also been reported to be involved in extrinsic apoptosis by inhibiting the interaction between CD95 and death domain-associated protein 6 (Daxx), an adaptor protein between CD95 and MAP kinase kinase kinase (MAPKKK) termed apoptosis signal-regulated kinase 1 (ASK1) [117,118,119,120,121]. In fact, ASK1 is reported to activate the Jun NH2-terminal kinase (JNK) pathway, which incites apoptosis [121]. However, when overexpressed, c-FLIP_L_ but not c-FLIP_S_ precludes the Daxx–CD95 interaction and thereby inhibits JNK activation and apoptosis [117,118]. It has been noted that JNK induces apoptosis by inducing activator protein 1 (AP-1) activation, which in turn induces apoptosis by promoting the expression of proapoptotic proteins such as TNF-α, CD95L, or via JNK-mediated phosphorylation of Bcl-2 homologous antagonist/killer (Bak), Bcl-2-like protein 11 (Bim), and p53 proteins [117,119,121,122,123,124,125,126]. Another possible explanation for JNK-induced apoptosis might be that the phosphorylation of p53 at S6 by JNK protects the ubiquitin-mediated degradation of p53, which in turn promotes apoptosis by increasing the transcriptional activation of the Bcl-2-associated X protein (Bax), Bak, the P53-upregulated modulator of apoptosis (Puma), nutrient-deprivation autophagy factor-1 (Noxa), and CD95 [127,128,129,130,131,132,133]. Within these lines, it is highly likely that JNK-induced apoptotic pathways involve the p53-mediated upregulation of proapoptotic proteins. Whatever the reason, the impairment of CD95–Daxx interactions by means of c-FLIP_L_ protects cells from apoptosis.

### 2.4. CD95-Mediated Complex II Formation

Emerging evidence has shown that besides DISC, CD95 stimulation drives a second signaling complex, i.e., complex II. It is formed within minutes after CD95 stimulation and contains procaspase-8a/b, c-FLIP_L/S/R_, FADD, and RIPK1 but not CD95 [134,135]. The mechanism leading to the formation of this complex is still elusive, but one of the hypotheses is that after reaching a certain size, DED filaments dissociate from the DISC to form complex II in the cytosol [33]. It is, however, clearly established that inhibitors of apoptosis proteins (IAPs) suppress the complex II formation [135]. Accordingly, the loss of cIAPs was shown to promote RIPK1-mediated complex II formation, which was named ripoptosome [135]. Furthermore, the ripoptosome has been shown to induce necroptosis upon the presence of RIPK3 in the cell as well as caspase inhibition. Under these conditions, this complex comprises RIPK3 and is termed necrosome [135,136]. Moreover, it has also been shown that CD95 stimulation leads to the formation of intracellular platforms, leading to the induction of anti-apoptotic pathways such as NF- κB. This platform was termed FADDosome and comprises FADD, RIPK1, c-FLIP, caspase-8, and the components of the IKK complex [137]. This in turn allows the induction of the NF-κB pathway. Importantly, the type of signal from complex II seems to be controlled by the ratio of caspase-8 and c-FLIP [138]. The role of cytosolic platforms based on RIPK1 in the transduction of apoptotic versus non-apoptotic signals is the subject of ongoing research.

### 2.5. Type I and Type II Cells in CD95 Apoptotic Signaling

There are two types of CD95-mediated apoptotic signaling, classified as type I and type II. The method of signal transduction depends on the particular cell type (Figure 1). Type I cells can activate high amounts of caspase-8 at the DISC to accomplish apoptosis [93,116]. In contrast, in type II cells such as hepatocytes, pancreatic beta cells, and most cancer cells, the apoptotic signal requires amplification due to the low amount of caspase-8 generated at the DISC [93,116,139,140]. Specifically, the BH3-interacting domain death agonist (Bid), a Bcl-2 family protein, is cleaved by caspase-8 to generate truncated Bid (tBid), which translocates to the mitochondrial extracellular membrane [116,141,142,143]. At the mitochondria, tBid is suggested to interact with cardiolipin, which mediates mitochondrial outer membrane permeabilization (MOMP) by promoting Bax or Bak oligomerization, followed by the release of cytochrome C, a second mitochondria-derived activator of caspase (SMAC)/direct IAP-binding protein with low PI (DIABLO), and high-temperature-requirement protein A2 (HTRA2)/omi stress-regulated endoprotease (OMI) into the cytosol [144,145]. Subsequently, cytochrome C, apoptosis protease-activating factor 1 (APAF-1), and procaspase-9 form the apoptosome, which serves as a platform for caspase-9 activation [141,146,147]. Ultimately, active caspase-9 in turn activates effector caspases-3 and -7, leading to apoptotic cell death [140,141,147]. As might be expected, both in type I and type II cells, CD95 stimulates the mitochondria-mediated cell death pathway. However, only type II cells require the amplification of apoptotic signaling because they contain higher X-chromosome-linked inhibitor of apoptosis protein (XIAP) levels, which block caspase-3 and -7 activity [148,149,150,151,152,153]. Furthermore, XIAP also was shown to degrade caspase-3 through ubiquitylation [149]. In line with this statement, genetic ablation of XIAP in type II cells reportedly changed their phenotype to that of type I cells [148]. In the same vein, the release of SMAC and HTRA2 was shown to detach XIAP from effector caspases, restoring the apoptotic signal [154,155,156,157]. Furthermore, type II cells depend on tBid-induced apoptotic signal amplification in contrast to type I cells [93,111,158].

Some studies have attributed the differences between type I and type II cells to the distinct CD95 localization in lipid rafts, characterized by the presence of high amounts of sphingolipids, cholesterol, and low-density lipoprotein, [98,148,159,160,161,162,163]. Furthermore, several studies have indicated that the lower caspase-8 activity at the DISC of type II cells might be attributed to differences in the composition of this complex. Specifically, higher phosphoinositide 3-kinase (PI3K) activity was detected in type II cells. It was suggested that PI3K activation excludes CD95 from lipid rafts [164,165]. Further, the phosphatase and tensin homologue (PTEN), an inhibitor of PI3K signaling, was observed to be downregulated in type II cells, leading to enhanced Akt activation [166,167]. Furthermore, in type II cells, increased astrocytic phosphoprotein PEA-15 phosphorylation at S116 was shown, the phosphorylation of which promotes the FADD–PEA-15 interaction and thereby inhibits CD95-mediated DISC formation [168,169,170]. In contrast, PI3K-downmodulator edelfosine, an ether lipid used as an antitumor drug, is reported to induce apoptosis by redistributing CD95 into lipid rafts [171]. Similarly, blocking PI3K using specific inhibitors such as LY294002 or wortmannin promotes CD95 clustering [164].

### 2.6. Dynamics of CD95 DISC

Recent years have seen remarkable advances in our understanding of DISC dynamics. Upon CD95 stimulation, DISC is formed within seconds [29,172]. Initially, it was supposed that at the DISC, c-FLIP would compete with procaspase-8 to bind directly to the DED of FADD [116]. However, recent studies have shown that c-FLIP binds to procaspase-8 within DED chains, which in the case of short c-FLIP isoforms, leads to the termination of DED chain elongation [32,36,94]. On the the hand, it was also reported that c-FLIP can bind to FADD as well as displace procaspase-8 from the FADD complex [35,94]. Further, it was suggested that FADD DED has distinct binding sites for c-FLIP and procaspase-8, suggesting that caspase-8 does not directly compete with c-FLIP isoforms for binding to FADD [115,172].

Stimulation strength is critical to the composition of the DISC and the type of signal. As highlighted above, CD95 stimulation leads to the induction of both apoptotic and non-apoptotic signaling. The competition of these two pathways determines the cell’s fate. High stimulation strength leads to the death of the cell, while the threshold stimulation strength might result in survival [54]. In fact, depending on the amount of stimulated CD95, the number and proportions of FADD, procaspase-8, -10, or c-FLIP in the DISC may alter. In particular, low CD95 stimulation strength results in longer DED filaments with relatively higher amounts of c-FLIP, leading to a rather low rate of apoptosis [33]. The latter was shown using quantitative mass spectrometry (MS) analyses [33]. In contrast, upon high stimulation strength, DISC contains shorter DED filaments with relatively lower amounts of c-FLIP [33,46,54,65,82]. In this scenario, the decreased number of c-FLIP in DED filaments upon high stimulation may be attributed to the increased number of active receptors, as the absolute concentrations of c-FLIP proteins are much lower than those of procaspase-8. One could argue that the generation of longer filaments under the low stimulation conditions may compensate for the low number of activated CD95 by providing more sites for procaspase-8 activation [33]. However, it cannot be ruled out that an increased amount of c-FLIP, particularly c-FLIP_S/R_, may limit procaspase-8 activation [54]. It has to be noted that regardless of the stimulation strength and length of DED filaments, procaspase-8 remains the major component of the DISC, exceeding the amount of FADD, procaspase-10, and c-FLIP several-fold [33]. These data strongly hint that differences in the composition of DISC may account for the differential outcomes of CD95 signaling. Indeed, upon low stimulation strength, procaspase-8 was shown to be processed only to p43/p41, and no further cleavage was detected after the first cleavage of procaspase-8 [65]. In this event, without the second-step cleavage of procaspase-8, CD95 signaling results in cell survival rather than apoptosis [101]. It is also important to note that CD95 stimulation upregulates the anti-apoptotic proteins B-cell lymphoma-extralarge/Bcl-2-like protein 1 (Bcl-X_L_), c-FLIP_R/S_, and p22-FLIP [68]. In contrast, upon high stimulation, procaspase-8 is fully processed, thereby active caspase-8 heterotetramer is generated [65]. Taken together, the stimulation strength of CD95 alters DISC stoichiometry, which is critical to caspase-8 activation [33,54]. A point to keep in mind is that c-FLIP proteins determine the composition of DED filaments and thereby act as a guardian of the threshold of CD95-mediated apoptosis.

Another caspase activated at the DISC is procaspase-10, a homolog of caspase-8. Even though it shares many substrates with caspase-8, it cannot substitute caspase-8 [173]. The role of caspase-10 at the DISC is not clear as yet, and it is still elusive whether caspase-10 can initiate apoptotic cell death in the absence of caspase-8. Interestingly, the ratio of caspase-10 to caspase-8 at the DISC is only one to ten, which might provide an explanation of why caspase-10 cannot efficiently initiate apoptosis [33]. Recently, it has been shown that independently of c-FLIP, caspase-10 impedes caspase-8 activation by reducing its DISC association, switching CD95 signaling from apoptosis to cell survival [174]. Consistent with this, the knockdown of caspase-10 is reported to sensitize HeLa and SK-Mel melanoma cells to CD95-mediated apoptosis [174]. Strikingly, the scaffold function of caspase-8 is reported to be crucial for caspase-10 recruitment to the DISC [174]. Accordingly, along with caspases-8, caspase-10 recruitment was shown to be downregulated in FADD-deficient Jurkat cells [175]. Given that caspase-8 promotes the further recruitment of c-FLIP as well as caspase-10, it is highly likely that caspase-8 at the DISC plays a central role by serving as a scaffolding protein for the proper activation of DED filaments [174].

#### 2.6.1. Modulation of DISC Dynamics with Posttranslational Modifications

Post-translational modifications (PTMs) of core DISC components also contribute to DISC dynamics. PTMs such as phosphorylation, glycosylation, ubiquitylation, palmitoylation, nitrosylation, methylation, glutathionylation, and SUMOylation of DISC constituents were shown to modulate CD95 signaling [176,177,178,179]. These PTMs represent additional checkpoints of CD95 signaling that may confer CD95 to act both as an apoptosis inducer and a cell-survival promotor.

##### PTMs of CD95 Influence DISC Dynamics

The phosphorylation of receptors is one of the key regulatory mechanisms in signal transduction. Interestingly, like other receptors, CD95 is shown to be reversibly phosphorylated by oncogenic Src family kinases (SFKs), in particular by Src and Yes [180,181]. Particularly, phosphorylations of CD95 at Y232 and Y291 in DD reportedly inhibit FADD recruitment and thereby block proapoptotic signals [180,182,183]. Further, phosphorylation at Y291 was shown to prime clathrin-dependent CD95 endocytosis and cell survival [180]. In support of this, the internalization of mutant non-phosphorylatable CD95 (Y291F) was defective [184]. Within these lines, enhanced CD95 phosphorylation has been shown to increase the resistance to CD95-induced apoptosis in several types of cancer [180,185]. Given that phosphorylated CD95 is translocated to the nucleus where it promotes the expression of cyclin D1 via the CD95/EGFR/STAT3 complex, it is highly likely that the phosphorylation of CD95 at Y291 induces prosurvival signals by means of cyclin D1 [182]. In addition to this, SFKs also phosphorylate the regulatory p85 subunit of PI3K, which in turn inhibits CD95 aggregation by excluding CD95 from lipid rafts [186]. Moreover, the dephosphorylation of CD95 by Src homology domain 2 (SH2)-containing tyrosine phosphatase-1 (SHP-1) was shown to promote CD95-mediated apoptosis [180,187,188,189]. In general, the phosphorylation of CD95 DD turns off the proapoptotic signal and turns on the prosurvival signal.

Similar to phosphorylation, glycosylation is also a highly dynamic and inducible process with huge potential to create regulatory networks [190]. CD95 has a molecular mass of about 35 kDa; however, it is mostly expressed as a glycosylated protein with a molecular mass ranging from 45 kDa to 54 kDa [12,13]. A series of elegant experiments showed that the extracellular domain of CD95 contains two N-glycosylation sites, N118 and N136, which contribute to CD95L binding and CD95 signaling [10,11,176,191]. Interestingly, a different glycosylation pattern of CD95 was shown between type I and type II cells. Specifically, type I cells reportedly have two major glycoforms of CD95 with different molecular masses, whereas type II cells mostly have one major CD95 form [10]. Thus, the presence of specific carbohydrate structures at CD95 may contribute to the generation of higher amounts of DISC in type I cells. Moreover, abrogation of the apoptotic signal due to defective CD95 oligomerization in T cells has been attributed to abnormal CD95 glycosylation [191]. The current advances in our knowledge indicate that the glycosylation of CD95 may provide fine regulation in the initiation of death signals; however, the association between the glycosylation pattern of CD95 and the strength of CD95 signaling still has to be uncovered.

Receptor clustering into higher-order structures is required for efficient apoptotic signaling. It has been shown that translocation into lipid rafts is crucial for effective CD95 oligomerization. Specifically, palmitoylation of CD95 at C199 in human cells and at C194 in murine cells was reported to be indispensable for its translocation into lipid rafts [160,177]. Accordingly, the C199V mutant CD95 in human HEK293T cells was defective in the localization to lipid rafts without affecting its interaction with FADD [192]. Moreover, primary B and T cells deficient in palmitoylation were shown to be resistant to CD95-mediated apoptosis [192]. These results confirm that the primary function of CD95 palmitoylation appears to support receptor clustering by targeting CD95 into lipid rafts for the efficient induction of apoptosis rather than to promote FADD recruitment. Surprisingly, despite defective apoptosis, the presence of C194V mutant CD95 was sufficient to prevent auotoimmunity in mice, which raises questions about the notion that CD95 prevents autoimmunity by inducing apoptosis [23,192].

The short-lived radical nitric oxide (NO) has emerged as a potent modulator of apoptosis. Similar to palmitoylation, S-nitrosylation of CD95 was also shown to be essential for its translocation into lipid rafts. Interestingly, CD95 nitrosylation occurs both at C199 and C304; however, nitrosylation only at C304 and not at C199 is reported to be involved in CD95 aggregation and DISC formation [193,194]. In spite of the fact that CD95 nitrosylation promotes CD95-mediated signaling strength by promoting receptor aggregation, the nitrosylation of caspases impairs their activation and consequently attenuates CD95-mediated extrinsic cell death via nitrosylation [195,196,197,198,199]. Due to the limited studies conducted on the nitrosylation of DISC components and other caspases, little is known about the modulation of CD95-mediated apoptosis by nitrosylation.

In cells undergoing oxidative stress, cysteine residues of proteins are oxidized by reactive oxygen species (ROS). The oxidation of cysteine residues may result in the generation of irreversible disulfide bridges and consequently impair the function of the proteins. Hence, proteins can function properly with only their cysteines in a reduced state. Glutathione protects proteins against irreversible oxidation by forming reversible disulfide bridges and thereby maintains thiol groups in a reduced state [200]. Specifically, a disulfide bridge is generated between the cysteine of glutathione and the cysteine of a protein, called S-glutathionylation [201,202]. Extensive work concerning further PTMs of CD95 has brought to light that mouse CD95 is S-glutathionylated in its DD at C294, augmenting apoptosis by inducing its distribution into lipid rafts [201,203]. These data indicate that the S-glutathionylation of CD95 may be an attractive target for therapeutic interventions, but further studies on CD95 S-glutathionylation are needed for a deeper understanding.

##### PTMs of FADD Influence DISC Dynamics

As an essential adaptor protein, FADD drives the generation of DISC, which is a principal site of action in the regulation of apoptotic or cell-survival signals. Studies on the modification of FADD have determined that this pivotal signaling DISC component is phosphorylated. In fact, FADD is modified at S194 (S191 of murine FADD), S200, and S203 by a number of kinases that result in its nuclear localization [204,205,206,207]. Strikingly, phosphorylation at S203 by mitotic kinase Aurora-A (Aur-A) is reported to prime FADD for phosphorylation at S194 by polo-like kinase 1 (Plk1) [204]. Since phosphorylation sites are localized outside of the DD and DED regions, the phosphorylation of FADD is reported to not interfere with DISC formation [29,208]. Overall, the function of FADD phosphorylation has not been elucidated yet precisely, but it is suggested that FADD phosphorylation contributes to the cell cycle rather than to DR signaling.

Apart from phosphorylation, another PTM observed on FADD is the glycosylation carried out by bacterial glycosyltransferases, which is described in detail in [209,210,211,212]. Importantly, intracellular glycosylation of the other components of the DR system such as CD95 also plays an important role in apoptotic and non-apoptotic signaling. Furthermore, the glycosylation of FADD is reported to inhibit the proinflammatory responses and apoptosis of host cells, thereby promoting the bacterial colonization of enterocytes [213]. In this context, it is conceivable that via the glycosylation of FADD, certain bacteria evade host immune defenses. However, it has to be noted that no endogenous FADD-modifying glycosyltransferase in eukaryotic cells has been determined yet.

Ubiquitylation also plays a major role in CD95 signaling control [214]. The addition of K48-linked ubiquitin chains to the protein leads to a signal for proteasome-mediated degradation, whereas K63-linked chains regulate the function of proteins [178]. FADD has been shown to be ubiquitylated by makorin ring finger protein 1 (MKRN1), an E3 ligase, as well as by the C terminus HSC70-interacting protein (CHIP). The MKRN1-mediated ubiquitylation type and site(s) of ubiquitylation on FADD are not known yet, but this ubiquitylation results in FADD degradation and, thereby, the downregulation of extrinsic apoptosis [215]. On the other hand, CHIP has been shown to induce K6-linked ubiquitylation of FADD within DD at K149 and K153 [216]. Although ubiquitylation at K149 and K153 has no particular effect on the DD interactions between FADD and CD95, ubiquitylation at these sites reportedly restrains CD95 DISC formation [216]. In line with this, increased DISC formation was shown in CHIP-depleted cells, showing higher sensitivity toward CD95-mediated cell death [216]. Similarly, mutations at the sites of ubiquitylation, at K149 and K153, promoted CD95L-induced apoptotic cell death [216]. Taken together, the ubiquitylation of FADD appears to be an important checkpoint in the decision of life and death upon CD95 stimulation.

SUMOylation has also been shown to control the CD95 pathway. The small ubiquitin-related modifier (SUMO) has been shown to modify multiple lysine residues within FADD. In fact, under the excessive calcium overload caused by calcium ionophore A23187 or by ischemic damage, FADD has been shown to be SUMOylated at K120, K125, and K149, but not under physiological conditions [217]. Interestingly, the SUMOylation of FADD was shown to induce its translocation to the mitochondria where it interacts with dynamin-related protein 1 (Drp1) and caspase-10, resulting in mitochondrial fragmentation and necroptosis [217]. Accordingly, MEFs expressing SUMOylation-defective FADD were reported to undergo significantly less mitochondrial fragmentation under calcium overload [217]. Given that excessive calcium influx is frequently observed under ischemic conditions and FADD SUMOylation is crucial during necrosis under a calcium overload, obtaining further insights into novel molecular mechanisms is worthy of investigation, which may provide new therapeutic modalities in ischemic injury [218].

##### PTMs of Caspase-8 Influence DISC Dynamics

Eight isoforms of procaspase-8 have been reported [219]. Procaspase-8a, a 55.7 kDa protein of 496 amino acids, and procaspase-8b, a 53.7 kDa protein comprising 479 amino acids, are two procaspase-8 isoforms predominantly recruited to the DISC [3,33,34,219,220,221]. Despite its activation displaying the onset of cell death, procaspase-8 is reported to be upregulated in many cancer cells. Accordingly, in response to CD95 activation, caspase-8 is found to trigger the expression of anti-apoptotic proteins, indicating alternative non-apoptotic, prosurvival functions of this apical caspase [222,223,224]. In this context, following epidermal growth factor (EGF) stimulation, procaspase-8 was shown to be phosphorylated by Src kinase at Y380 in the 10-amino acid linker region between the two catalytic subunits (i.e., p18 and p10) [222,225,226,227]. Since Y380 is located on the critical side for proteolytic cleavage, the phosphorylation of Y380 impairs cleavage and consequently prevents the complete maturation of caspase-8 by blocking the release of the p18 subunit from the DISC [222]. Moreover, the inhibition of p18 release impairs the further cycling and release of active catalytic subunits into the cytosol, thereby impeding the downstream activation of effector caspases and attenuating CD95-mediated apoptosis [222]. It is also important to highlight that besides Src kinase, the phosphorylation of procaspase-8 at Y380 can also be carried out by other members of SFKs such as Fyn and Lyn [221,226,228]. Further, dephosphorylations of caspase-8 at Y380 and Y465 by SHP-1 were shown to promote caspase-8 activity and apoptosis [221]. Another phosphorylation on caspase-8 is carried out by Lyn at Y465, which renders resistance to its activational cleavage and thereby inhibits apoptosis [221]. In support of this, increased Lyn activation reportedly increases resistance to apoptosis [229,230,231]. As might be expected, increased caspase-8 and -3 activities are observed in cells with Y465F mutant procaspase-8 [221]. Collectively, the phosphorylation of procaspase-8 at Y380 and Y465 inhibits extrinsic apoptosis by interfering with caspase-8 activation. In addition, given that the Src kinase also phosphorylates PI3K and thereby excludes CD95 from lipid rafts, it is highly likely that EGF precludes CD95-mediated apoptosis in two different ways: either by promoting caspase-8 phosphorylation or by excluding CD95 from lipid rafts [186].

Extensive work concerning caspase-8 phosphorylation revealed further phosphorylation sites on caspase-8. For example, in neutrophils, p38 MAPK was shown to phosphorylate caspase-8 at S364, which protects these cells from CD95-mediated apoptosis by inhibiting the activity of the p18 large subunit [232,233]. In parallel, active ERK1/2 (pERK 1/2) as well as cyclin-dependent kinase 1 (CDK1) reportedly phosphorylate procaspase-8 in the p10 subunit at S387, thus inhibiting the further activation of caspase-8 and thereby shielding cells against extrinsic apoptosis during mitosis [228,234,235]. Consistent with this, elevated activation of ERK signaling has been detected in various human tumors, protecting cancer cells from undergoing apoptosis in the presence of CD95L [236,237]. Additionally, CDK1-mediated phosphorylation at S387 is reported to prime procaspase-8 for interaction with Plk1, which in turn phosphorylates the initial caspase at S305 and thereby inhibits its processing [238]. In contrast, polo-like kinase 3 (Plk3)-induced phosphorylation at T273 has been reported to promote the activation of caspase-8 [239]. In addition to these, ribosomal S6 kinase 2 (RSK2)-induced phosphorylation of caspase-8 at T263 was shown to promote its ubiquitylation and proteasomal degradation of caspase-8 [240]. Taking into account that phosphorylation generally impairs apoptotic signaling, it is no surprise then that caspase-8 phosphorylation has been shown to be upregulated in several human cancers [225,241,242].

Along with the well-established role of caspase-8 phosphorylation in the modulation of CD95 signaling, one recent study clearly documented that the modification of caspase-8 with a single carbohydrate molecule, N-acetylglucosamine, impairs the activation of caspase-8. In fact, caspase-8 is shown to be O-GlcNAcylated at the cleavage sites and thereby blocks its activation upon induction of apoptosis by TNFα [243]. Furthermore, increased caspase-8 O-GlcNAcylation upon Thiamet-G treatment, an O-GlcNAcase (OGA) inhibitor, further downregulated the cleavage of this initial caspase without altering its dimerization [243]. However, the comprehension of the mechanisms of the role of O-GlcNAcylation in caspase-8 activation still remains unexplored. Thus, whether caspase-8 is glycosylated upon CD95-stimulation has not yet been unraveled, and therefore, the glycosylation site(s) remain to be discovered.

In addition to the phosphorylation and glycosylation of caspase-8, CD95-mediated apoptosis is also modulated by means of ubiquitylation. In this regard, DISC-associated caspase-8 has been shown to be modified by TNF receptor-associated protein 2 (TRAF2)-dependent K48 ubiquitylation at lysines 224, 229, and 231 in the large subunit [244]. Ubiquitin tagging extinguishes caspase-8 activity by destining them to rapid proteasomal degradation that protects cells from apoptosis [244]. In line with this statement, the overexpression of TRAF2 in pancreatic cancer cells was shown to protect these cells from CD95-mediated apoptosis, while the downregulation of TRAF2 enhanced extrinsic apoptosis [244,245,246]. In the same vein, increased TRAF2 recruitment to the caspase-8/c-FLIP heterodimer has been reported to enhance the NF-κB pathway [247,248,249]. Several studies have established that p43-FLIP is associated with TRAF2 and RIPK1, which suggests another way for NF-κB activation [33,247,248,250]. Similar to K48 ubiquitylation, procaspase-8 is K63-linked ubiquitylated at K215 by homologous to the E6-AP carboxyl terminus 3 (HECTD3), which was also shown to inhibit caspase-8 activity [251,252]. In contrast to ubiquitylation carried out by TRAF2 or by HECTD3, Cullin-3 (Cull-3)-driven K63 ubiquitylation on the p10 subdomain at K461 augments the apoptotic signaling pathway by stabilizing the active caspase-8 heterotetramer [178]. Additionally, upon tunicamycin-induced endoplasmic reticulum (ER) stress, Tripartite Motif-Containing Protein 13 (TRIM13) reportedly promotes caspase-8 activity by conjugating caspase-8 with K63-linked ubiquitylation, the modification site(s) of which has not been revealed yet [253]. Thus, ubiquitylations at different sites of caspase-8 appear to keep each other in check. Considering that TRAF2 is also involved in ERK activation, resulting in the inhibition of caspase-8, it is rational to suppose that TRAF2 sets a critical barrier for DR-mediated apoptosis by directly tagging caspase-8 with ubiquitin or phosphorylating caspase-8 by means of ERK [234,254,255].

The other reported PTM of caspase-8 is nitrosylation, which reportedly impairs its activity and apoptosis [198]. In spite of the fact that nitrosylation inhibits caspase-8 activity, no information is available yet about the nitrosylation site(s) on caspase-8. In this sense, further future studies are needed to figure out this ignored issue. Given that an infusion of NO donors may limit caspase-8 activation, the modulation of the extrinsic apoptotic pathway via caspase-8 S-nitrosylation may be a promising novel strategy. However, our understanding of the role of S-nitrosylation in apoptosis is fragmented, therefore further studies are needed for a deeper understanding.

Another important modulator of caspase-8 is E3 SUMO-protein ligase PIAS1, which SUMOylates caspase-8 at K156 in DED2, leading to an increase in caspase-8’s molecular weight of 20 kDa [256]. Interestingly, SUMOylated caspase-8 (p75) was found to be mostly located in the nucleus of the cells, suggesting that caspase-8 SUMOylation is associated with its nuclear localization [256]. Moreover, SUMOylation reportedly does not interfere with the processing of caspase-8 even in the nucleus, where it might cleave other undefined specific nuclear substrates [256]. One recent study revealed that nuclear caspase-8 inhibits CDK9 activation and consequently modulates RNAPII-mediated global transcription, including cell migration genes [257]. Due to the very limited information available, little is known about the role of caspase-8 SUMOylation in CD95 signaling. Therefore, we cannot rule out other potential roles of caspase-8 SUMOylation in CD95 signaling.

##### PTMs of c-FLIP Influence DISC Dynamics

The PTMs of c-FLIP play also a pivotal role in the DISC dynamics and the type of signaling induced. Several c-FLIP phosphorylation sites, such as S4, T166, S193, Y211, and S273, have been identified to date [258,259,260,261]. As the phosphorylation of c-FLIP at T166 resides on the FADD interacting side, it is rational to suppose that phosphorylation on this side may prevent the c-FLIP/FADD association [115,262]. However, in contrast to this prediction, phosphorylation at T166 does not interfere with the interaction between these two DISC proteins [115,262]. Nevertheless, the phosphorylation on this side is reported to be a prerequisite for K48 ubiquitylation at the succeeding K167, which destines c-FLIP_L_ to proteasomal degradation [259,261,263]. Similarly, phosphorylation at S4 and Y211 in c-FLIP_S_ as well as at S273 in c-FLIP_L_ was shown to facilitate the ubiquitylation and protosomal degradation of c-FLIP isoforms [259,260,264]. In contrast, phosphorylation at S193 blocks the ubiquitylation of c-FLIP at K192 and K195, which increases the stability of c-FLIP_S_ but not c-FLIP_L_ [258,265]. As S193 resides outside DED, as might be expected, S193 phosphorylation was shown to not affect c-FLIP’s DISC-binding affinity [258].

The available evidence suggests that a covalently attached phosphate may cause conformational changes in c-FLIP isoforms and alter the outcomes of DR signaling. In line with this statement, the phosphorylation of c-FLIP_L_ by calcium/calmodulin-dependent protein kinase II (CaMKII) has been shown to promote c-FLIP recruitment to the DISC that renders glioma cells resistant to CD95-mediated apoptosis [264]. Strikingly, only the phosphorylated c-FLIP_L_ and its cleavage product phospho-p43-FLIP were detected at the CD95 DISC in CH-11-resistant glioma cells [264]. Interestingly, phosphorylated p43-FLIP has been shown to block the second cleavage step of caspase-8 that switches CD95 signaling from cell death to proliferation [264]. Along with the well-established role in c-FLIP recruitment to the DISC, CaMKII also transcriptionally upregulates c-FLIP expression [266]. With this in mind, the inhibition of CaMKII enzymatic activity may induce apoptosis in two ways: either by depleting c-FLIP expression or impeding c-FLIP DISC recruitment. Consistent with this, the pharmacological inhibition of CaMKII enzymatic activity has been demonstrated to downregulate c-FLIP expression, which promotes sensitivity to DR-induced apoptosis [264,267].

The turnover of c-FLIP is strongly regulated by its ubiquitin-dependent degradation rate [268]. Due to the different ubiquitylation mechanisms for the long and short isoforms, the pathways regulating c-FLIP levels in the cell are surprisingly complex. It is well known that due to the unique C-terminal tail, c-FLIP_S_ is more prone to ubiquitin-dependent degradation [265]. In particular, FLIP_S_ has been shown to be ubiquitylated by E3 ubiquitin ligase atrophin-interacting protein 4 (AIP4) and the human orthologue of E3 ubiquitin-protein ligase Itchy homolog (ITCH) [269]. Moreover, c-FLIP_S_ was shown to be ubiquitylated at K192 and K195, leading to the rapid degradation of this short isoform [265]. Taking into consideration that K192 and K195 are also present in c-FLIP_L_, it is logical to assume that c-FLIP_L_ can also be ubiqitylated. In contrast, studies with mutant c-FLIP_L_ have indicated that the principal ubiquitylation sites are distinct in c-FLIP_S_ and c-FLIP_L_ [265]. Thus, c-FLIP_L_ was shown to be ubiquitylated at a different lysine residue, i.e., at K167. As mentioned above, for the ubiquitylation at this site of c-FLIP_L_, phosphorylation of the preceding amino acid (i.e., T166) is a prerequisite [261]. Consistent with this statement, cells with T166A and K167R single and double mutants have shown increased FLIP_L_ levels [261]. Previous work by Chang et al. provided information that ITCH ubiquitylates and degrades c-FLIP_L_, providing yet another mechanism for the regulation of c-FLIP levels [263]. In addition, c-FLIP_L_ was shown to undergo M1 linear ubiquitylation by means of E3 ubiquitin-protein ligase RNF31, a component of the linear ubiquitin chain assembly complex (LUBAC). In fact, M1-linked ubiquitin is reportedly attached to K351 and K353 in the p20 subunit of c-FLIP_L_ and consequently protects this protein from proteasomal degradation [270]. Collectively, insight into this delicate balance between different molecular mechanisms in the regulation of c-FLIP expression may open new horizons for the development of therapeutics to overcome the apoptotic resistance observed in various malignancies.

The other reported PTM of c-FLIP_L_ is nitrosylation. Depending on the cell type and redox status, NO has been shown to act in both pro- and anti-apoptotic manners; however, the underlying mechanisms are still only partly understood [271,272]. The proapoptotic function of NO is attributed to its oxidative stress-inducing ability [271]. Additionally, the ligation of CD95 by its ligand was shown to induce NO generation, with a marked decrease in c-FLIP expression [272]. On the other hand, the exposure of lung epithelial cells to NO donors resulted in the nitrosylation of c-FLIP_L_ at C254 and C259, which blocked ubiquitylation and, consequently, the proteasomal degradation of c-FLIP_L_ [273]. As a consequence, the accumulation of FLIP_L_ within the cells results in an increase in resistance to CD95-mediated apoptosis [272]. Since short isoforms have none of these nitrosylation sites, NO might not play a role in the control of their expression.

## 3. CD95-Mediated Non-Apoptotic Signaling

Since the CD95 signaling system is generally appreciated for its role in inducing apoptosis, the non-apoptotic functions of CD95 remained underappreciated and, consequently, poorly investigated [3,98]. Nevertheless, based on the substantial evidence built up regarding non-apoptotic functions, CD95 is now recognized as a multifaceted receptor. Indeed, over the years, it has become clear that CD95 also elicits a number of cell death-independent signaling pathways including p38, ERK1/2, JNK1/2, and NF-κB pathways [13,25,63,274,275,276,277,278]. Non-apoptotic signaling pathways reportedly promote the chemotaxis of phagocytes toward apoptotic cells by generating the ‘find-me’ signal via the secretion of chemokines such as MCP-1 and IL-8 [279,280]. In addition, CD95 is reported to be involved in cell migration and neuron outgrowth [66,281,282]. Furthermore, CD95 signaling was shown to be essential for efficient liver generation following partial hepatectomy [283]. A variety of studies have demonstrated that the strength of the activation signal, and consequently the amount of c-FLIP at the DISC, determines the fate of CD95 signaling [13]. In this regard, recent studies have shown how high levels of c-FLIP switch the architecture of the DED filament to non-apoptotic by interrupting the formation of the procaspase-8 DED filament, leading to procaspase-8 dimerization [35]. Moreover, CD95 stimulation generates p43-FLIP, which in turn was reported to recruit TRAF2, RIPK1, and RAF proto-oncogene serine/threonine-protein kinase (RAF1), resulting in the activation of non-apoptotic signaling [110,248,284].

A number of studies have reported different outcomes of CD95/CD95L stimulation depending on various forms of CD95 and CD95L. Likewise, it was shown that membrane-bound CD95L induces apoptotic signaling and soluble ligand activation of NF-κB, which might lead to autoimmunity [83]. Furthermore, cells carrying heterozygous mutations in the CD95 DD were shown to be deficient in the induction of apoptosis but allowed the full activation of NF-κB [9]. Accordingly, compared to the wild-type CD95-expressing cells, much stronger NF-κB activation is reported in human and mouse cells carrying mutant CD95, indicating that CD95 stimulation below a certain threshold level induces cell survival rather than cell death [285]. Thus, the existence of one functional allele is only sufficient for non-apoptotic signaling, and the implementation of the canonical apoptotic signal requires two functional CD95 alleles [9]. It should be kept in mind that apoptotic and non-apoptotic pathways can be simultaneously activated in the presence of a strong activation signal. However, once CD95 is strongly activated, because of strong procaspase-8 processing, cells usually undergo apoptosis. Collectively, it is tempting to speculate that under these strong activation conditions, the apoptosis-inducing capacity of CD95 masks the NF-κB activation aptitude of this DR.

Based on its ability to deliver death signaling, CD95 was initially classified as a tumor-suppressor protein. However, despite being frequently downregulated during cancer progression, the complete loss of CD95 is rarely seen in human cancers [285,286]. A recent study showed that a complete loss of CD95 expression in cancer cells leads to an unconventional cell death program called “death induced by CD95 elimination, DICE”, which has yet to be further investigated [287,288]. Furthermore, in a phase II clinical trial, targeted inhibition of CD95 signaling by interfering with the CD95/CD95L interaction in relapsed glioblastoma patients reportedly reduced the invasiveness of glioma cells and prolonged patient survival [274,289]. Further, the knockdown of CD95 in numerous cancer cell lines resulted in a profound reduction in the growth of cancer cells [290]. There is compelling evidence that tumor cells require a baseline level of CD95 expression to survive and proliferate [18,186,286,290,291,292,293]. In support of this, the downregulation of CD95 in cancer cells is reported to impair apoptosis and the efficient elimination of malignant cells, while it behaves like a growth factor receptor in cancer cells, which is ascribed to low stimulation strength [186,290,294,295]. In parallel, a single-point mutation in one of the CD95 alleles was shown to convert CD95 from a tumor suppressor to a tumor promotor, a situation frequently observed in advanced human cancer [285,296,297,298,299,300,301,302,303]. All of these data prompted the opinion that CD95 is a multifunctional protein whose function is not limited to the stimulation of apoptosis, and in different pathophysiological contexts, it can even contribute to carcinogenesis [290,304,305]. There is also a scenario in which interactions between CD95 in immune cells and its ligand in cancerous cells may induce immune cell apoptosis and immunosuppression, conferring cancer cells a survival advantage [306,307,308]. Hence, careful consideration needs to be given to the fact that CD95 signaling may entail a not-yet-elucidated complex network with distinct outcomes [183,309]. Hence, without a comprehensive understanding of the molecular basis of non-apoptotic CD95 signaling, these therapies may face major challenges. The NF-κB pathway is considered a major anti-apoptotic pathway [44,137]. Since other cell survival mechanisms are largely beyond the scope of this review, in this section, we will focus mainly on CD95-induced NF-κB activation.

### General Aspects of NF-κB Activation

**Figure 2:**
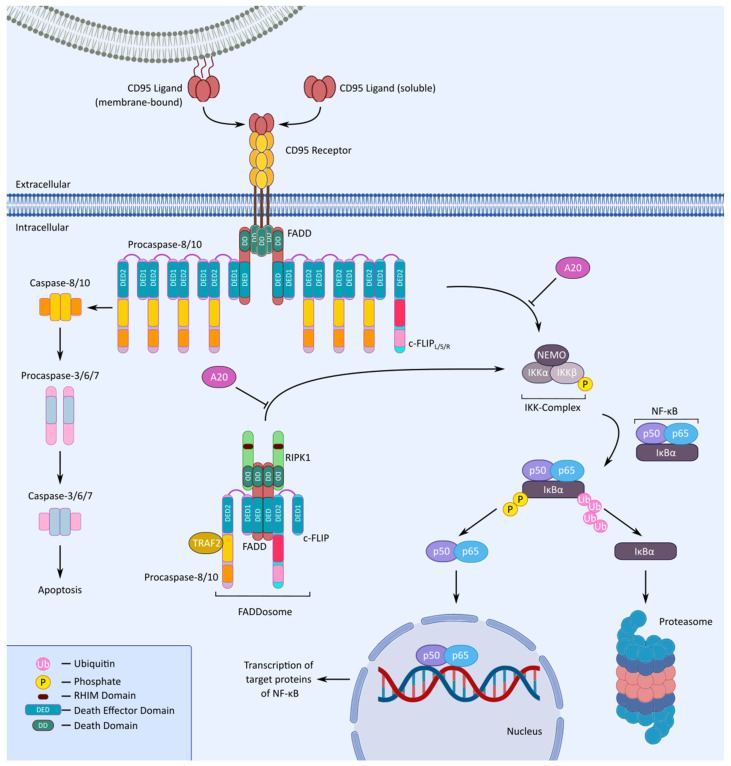
The scheme of CD95-mediated apoptotic and non-apoptotic signaling. Various factors contributing to the induction of apoptotic versus non-apoptotic NF-κB pathways are shown. CD95 stimulation leads to the formation of the DISC and the FADDosome, the latter consisting of FADD, RIPK1, c-FLIP, and caspases -8 and -10. Both the DISC and the FADDosome can have proapoptotic as well as antiapoptotic effects. DED proteins interact with NEMO, a part of the IKK complex. The interaction with NEMO results in the activation of IKK complex, subsequent ubiquitin-mediated proteasomal degradation of IκBα, and translocation of p50 and p65 into the nucleus, where they activate NF-κB target genes. A20 can inhibit activation of the IKK complex. Figure created using BioRender.com, accessed on 26 September 2024.

NF-κB is a transcription factor family composed of p65 (RelA), RelB, c-Rel, p50 (and its precursor p105), and p52 (and its precursor p100) that regulates the genes controlling apoptosis, cell survival, development, and immune responses [310,311,312,313,314]. In most cells, p65/p50 heterodimers are sequestered in the cytosol by inhibitors of the κB protein (IκB) [311]. The canonical NF-κB pathway is mediated by the activation of the IκB kinase (IKK) complex, composed of two catalytic subunits (IKKα and IKKβ) and a regulatory subunit (IKKγ/NEMO) [113,311,315,316,317,318]. DRs including CD95 were shown to activate the IKK complex, of which the IKKβ subunit in turn phosphorylates IκB, leading to its K48-linked polyubiquitylation and consequent degradation via the proteasome (Figure 2). Once IκBα is degraded, p65 is translocated to the nucleus where it initiates the transcription of several p65 target genes [312,315,316,317,318,319,320,321,322]. CD95-mediated NF-κB induction has been reported to activate the transcription of anti-apoptotic genes, resulting in the inhibition of apoptosis [77].

The NF-κB activation pathway has been best studied for tumor necrosis factor receptor 1 (TNFR1), the stimulation of which activates the IKK complex within seconds to a few minutes [323,324,325]. In recent years, CD95 has also been described to activate the NF-κB pathway [63,65,101,326]. However, compared to the TNFR1-mediated NF-κB pathway, upon CD95 stimulation, IKK complex activation and subsequent p65 nuclear translocation were shown to be considerably delayed [279]. Due to the very limited information available, the detailed molecular mechanism of CD95-mediated NF-κB activation has largely remained unclear. What is clear so far is that similar to TNFR1 signaling, RIPK1 also appears to be a central player in CD95-mediated NF-κB activation, indicating that CD95 may utilize similar machinery to TNFR1 to activate the NF-κB pathway [13,27,224,311,327,328,329]. Further experiments showed that different from TNFR1 signaling, FADD is essential for CD95-mediated NF-κB activation, demonstrating that despite some similarities, these two pathways may also have certain distinct activation mechanisms [328]. Accordingly, a deficiency of FADD resulted not only in the inhibition of CD95L-induced apoptosis but also impairment of the proliferation of T lymphocytes and Jurkat cells [330,331,332,333]. Along with FADD, caspase-8 is reported to be essential to inducing NF-κB activation in response to CD95L stimulation [50,224,328]. Interestingly, with its scaffolding function in the caspas-8/TRAF2/RIPK1 complex, procaspase-8 plays a critical role in the IKK complex and NF-κB activation, whereas active caspase-8 prevents sustained NF-κB activation by cleaving RIPK1 [16,81,98,174,224,246,248,250,329,334,335]. Within this line, caspase inhibitors were shown to enhance CD95-mediated NF-κB signaling [224,248,334]. CD95-induced NF-κB activation has been reported to have slower kinetics than that of TNF-α induced activation [328]. Furthermore, considering that CD95 induces the upregulation of TNF-α expression, it is highly likely that there are further links between classical and CD95-mediated NF-κB signaling pathways [336]. Thus, further studies are required to determine how important the crosstalk between TNF-α-mediated and CD95L-induced NF-κB signaling is. Increasing evidence indicates that CD95-mediated NF-κB activation and apoptosis occur in parallel [13]. Indeed, the stimulation of HeLa cells with CD95L resulted in IκBα phosphorylation and caspase-8 activation, indicating the simultaneous stimulation of NF-κB activation and apoptosis induction [27]. In this context, it is important to note that apoptosis and NF-κB pathways might mutually inhibit each other. Moreover, in CD95-sensitive cells, a loss of control in anti-apoptotic checkpoints could shift proliferative NF-κB signaling to death signaling.

Extensive work on CD95-mediated NF-κB activation revealed that c-FLIP proteins are important in NF-κB signaling and, depending on the expression level, c-FLIP isoforms can regulate NF-κB activation positively or negatively [13,43,50]. The overexpression of c-FLIP_L_ was shown to activate NF-κB by enhancing the recruitment of TRAF2 and RIPK1 into the CD95 DISC [337,338]. It has to be noted that not c-FLIP_L_ itself but rather the cleavage products of c-FLIP_L_, p43-FLIP, and p22-FLIP were reported to activate the NF-κB pathway [75,113]. In line with this, no NF-κB activation was detected in cells with non-cleavable c-FLIP_L_ [248]. Specifically, one of the suggested hypotheses is that p43-FLIP, a cleavage product of c-FLIP_L_ at D376, recruits TRAF2, which is an essential adaptor protein in NF-κB [247,248,284]. In contrast, another study revealed that non-cleavable c-FLIP_L_ (D376A) induces spontaneous NF-κB activation. Further, along with p43-FLIP, p22-FLIP, a cleavage product of c-FLIP_L_ at D196, also turned out to play a prominent role in the engagement of NF-κB signaling [3,13,93,99,113,248,311]. Of note is that, as D196 is localized between DED2 and the caspase-like domain, p22-FLIP can also be generated by the cleavage of short isoforms on this side [94]. Further studies revealed that p43-FLIP can be generated by the DISC-associated procaspase-8/c-FLIP_L_ heterodimer or mature caspase-8 heterotetramer (p10_2_-p18_2_) [99,110,113,114,115,116,334]. However, unlike p43-FLIP, studies on non-apoptotic primary T and B cells as well as mature dendritic cells revealed that p22-FLIP can be generated from procaspase-8/c-FLIP_L_ heterodimers [79,99,113]. Strikingly, for the generation of p22-FLIP, CD95 stimulation is reported to be dispensable [113,311]. In support of this statement, c-FLIP_L_ and procaspase-8 have been shown to form a DR-independent cytoplasmic complex, in which c-FLIP_L_ is cleaved to generate p22-FLIP [113]. Interestingly, both p22-FLIP and p43-FLIP were shown to interact with the IKK complex [75,113]. In fact, p22-FLIP is reported to be associated with the IKK complex via the regulatory subunit (IKKγ/NEMO), while p43-FLIP interacts with the catalytic subunit IKKα [13,113]. Similar to p22-FLIP, c-FLIP_S_ was also shown to interact with IKKγ and thereby can activate the NF-κB pathway [339,340,341]. Growing evidence has revealed that p43-FLIP is involved in the phosphorylation of the catalytic subunit IκBα, resulting in its degradation and subsequent translocation of p65 to the nucleus [13]. In support of this, p43-FLIP is detected in HeLa cells 20 min after CD95 stimulation, which correlates well with an increase in IκBα phosphorylation [311]. Collectively, these studies indicate that the processing of c-FLIP_L_ into p43-FLIP by caspase-8 is indispensable for CD95-mediated NF-κB activation.

NF-κB activation correlates to p43-FLIP levels, the generation of which decreases in the presence of both low and high c-FLIP_L_ levels [13,311]. In contrast, increased p43-FLIP generation has been reported upon intermediate c-FLIP_L_ levels [13]. This bell-shaped p43-FLIP generation profile may be explained by the fact that an increasing concentration of c-FLIP_L_ leads to an increase in the generation of p43-FLIP until it reaches a maximum, beyond which the level of p43-FLIP drops. This drop-off in p43-FLIP levels may be attributed to the inhibitory effect of c-FLIP_L_ on its high concentration of procaspase-8 activity. Further, as c-FLIP_L_ inhibits the recruitment of caspase-8 to the CD95 DISC, the inhibition of CD95-induced p43-FLIP generation and NF-κB activation from a certain point on may be ascribed to the inhibition of caspase-8 recruitment under c-FLIP_L_-overexpressing conditions [52,116]. In concert with these predictions, further studies confirmed that high levels of c-FLIP_L_ block procaspase-8 activity, whereas intermediate levels of c-FLIP_L_ promote caspase-8 activity by stabilizing the active center of procaspase-8 [54,55]. Along with this, the decrease in procaspase-8 levels significantly reduced p43-FLIP generation after CD95 stimulation [13]. Furthermore, caspase-8-deficient cells reportedly have defects in IKK activation and, consequently, in the activation of the NF-κB pathway [3,224]. Overall, the amount of c-FLIP_L_, as well as its ratio to procaspase-8, is critical in p43-FLIP generation. It also must be noted that the C-terminal domain of c-FLIP_L_ inhibits CD95-mediated NF-κB activation by impairing the interaction between caspase-8 and RIPK1 [50]. Thus, the generation of p43-FLIP and p12-FLIP promotes NF-κB activation synergistically where p43-FLIP recruits TRAF2, while the removal of p12-FLIP from c-FLIP_L_ facilitates the interaction of caspase-8 with RIPK1. Collectively, the initial concentrations of c-FLIPs as well as c-FLIP_L_ are the most important factors in the decision of whether cells will succumb to apoptosis or survive in response to CD95 apoptotic signaling.

The molecular mechanisms responsible for switching from apoptotic to non-apoptotic pathways remain largely enigmatic. As activated NF-κB upregulates the most important anti-apoptotic proteins such as c-FLIP, anti-apoptotic Bcl-2 family members, and XIAPs, these anti-apoptotic proteins also appear to be involved in switching from a death signal to a survival signal [94,116,342]. Further, zVAD-fmk, blocking the downstream signal from the DISC but not the generation of p43-FLIP, was shown to switch off apoptotic signaling and switch on NF-κB signaling [13,101,113]. Considering that the inhibition of effector caspases with zVAD-fmk does not inhibit NF-κB signaling, it is highly likely that apoptotic and NF-κB pathways already diverge at the DISC. A recent study revealed a novel interaction partner of CD95, Kip1 ubiquitination-promoting complex protein 2 (KPC2) [343]. Specifically, KPC2 was shown to interact with the C-terminal region of CD95 and serve as an adaptor to recruit RelA (p65) and KPC1, [343]. Eventually, an increase in the p50 subunit led to the formation of p65/p50 heterodimers that sequester p65 at the plasma membrane and thereby impair p65 nuclear translocation and NF-κB-driven gene expression. Accordingly, the loss of CD95 in human MDA-MB-231 and mouse 4T1 triple-negative breast cancer cells (TNBCs) was reported to result in IKKα/β phosphorylation, a hallmark of NF-κB activation [343].

Extensive work has revealed that an intracellularCD95 signaling complex, termed FADDosome, is formed upon CD95 stimulation, comprising FADD, RIPK1, caspase-8, and caspase-10 but not containing CD95 [74,224,344]. Caspase-8 has been reported to serve as a scaffold protein, the catalytic activity of which is not required for FADDosome formation [81,345]. Within FADDosome, RIPK1 and TRAF2 bind to the ubiquitin-binding domain of IKK in a K63 ubiquitin-chain-dependent manner, leading to the activation of NF-κB signaling (Figure 2). Interestingly, high concentrations of c-FLIP_L_ were shown to block FADDosome-mediated inflammation, which is in line with previous reports on high concentrations of c-FLIP proteins blocking DL-mediated NF-κB activity [138]. Consistent with this, the activation of CD95 in TRAF2-overexpressing cells resulted in enhanced NF-κB activation [245]. Moreover, it was shown that p43-FLIP can recruit RIPK1 and TRAF2 in the absence of CD95 [74,110]. The results of a recent study showed that the constitutive expression of p22-FLIP or c-FLIP_S_ leads to the formation of the c-FLIP/FADD/RIPK1 complex, resulting in NF-κB activation [340]. Thus, RIPK1 also appears to be an essential component in FADDosome-implicated NF-κB activation. In line with this, the deletion of RIPK1 reportedly blocks NF-κB activation [337]. Importantly, the presence of the C-terminal region of IKKγ/NEMO has been shown to be a prerequisite for c-FLIP_L_-, p22-FLIP-, or c-FLIP_S_-induced IKK kinase activation and, consequently, NF-κB signaling [340].

Overall, the ability of CD95 to deliver a death signal or stimulate cell survival depends on the cellular context. In addition to several other factors, initial c-FLIP levels, stimulation strength, and PTMs of various molecules of the CD95 signaling machinery can act as signal processors deciding between life and death.

## 4. Conclusions

CD95 is known as a DR prototype; however, the functions of CD95 are not limited to the induction of apoptosis. CD95 stimulation may also lead to necroptosis or even cell survival. The CD95-mediated apoptotic and non-apoptotic signaling pathways share many factors such as FADD, caspase-8, and c-FLIP (Figure 2). It might be paradoxical that the same machinery can be used by the cell to mediate apoptotic and non-apoptotic signaling pathways. The crosstalk of death and survival may occur at different levels of signal transduction, and various checkpoints have been reported to be involved in the outcome of CD95 signaling. In particular, the initial concentration of c-FLIP is the most important factor in the determination between death and survival signaling as it shapes the dynamics of the DISC. Another important factor is the dynamics of complex II assembly versus DISC and the type of signals, which can be induced by CD95-mediated intracellular complexes. The strength of activation signals, the form of CD95L, the degree of CD95 oligomerization, and the PTMs of CD95, FADD, procaspase-8, and c-FLIP_L_ are also important checkpoints in the decision between life and death. Furthermore, various proteins associated with CD95 DD in different cell types, particularly leading to the formation of DISC versus MISC, determine the various outcomes of CD95 stimulation. Additionally, the threshold for CD95 to stimulate NF-κB is much lower than to activate apoptosis, which provides the possibility of “bistability” behavior and both life and death outcomes within one population of cells. The molecular architecture of DED filaments also plays a key role in life/death decisions since the intricate stoichiometries of the DED proteins play a major role in determining cell fate upon CD95 stimulation.

Together, the function of CD95 as a DR corresponds to its “daily work”, while it acts as a “night killer” by promoting proliferative signals in certain cellular contexts. However, the factors influencing whether CD95 signaling induces apoptotic or survival signaling remain to be fully determined and analyzed in the future by systematic approaches.

## Data Availability

Not applicable.

## Abbreviations

AIP4, E3 ubiquitin ligase atrophin-interacting protein 4; AP-1, activator protein 1; APAF-1, apoptosis protease activating factor 1; ASK1, apoptosis sig-nal-regulated kinase 1; Aur-A, mitotic kinase Aurora-A; Bak, Bcl-2 ho-mologous antagonist/killer; Bax, Bcl-2 associated X protein; Bcl-xL, B-cell lympho-ma-extralarge/Bcl-2-like protein 1; Bid, BH3 interacting-domain death agonist; Bim, Bcl-2-like protein 11; CaMKII, calcium/calmodulin-dependent protein kinase II; CD95 (FAS/APO-1), cluster of differentiation 95; CD95L, CD95 ligand; CDK1, cyclin dependent kinase 1; c-FLIP, cellular FLICE inhibitory protein; CHIP, C terminus HSC70-interacting protein; CRD, cysteine-rich domain; Cull-3, Cullin-3; Daxx, death do-main-associated protein 6; DD, death domain; DED, death effector domain; DIABLO, direct IAP-binding protein with low PI; DICE, death in-duced by CD95 elimination; DISC, death-inducing signaling complex; DR, death receptor; EGF, epidermal growth factor; EPEC, enteropathogenic Esche-richia coli; ER, endoplasmic reticulum; FADD, Fas-associated protein with death domain; GalNAc, N-acetylgalactosamine; GlcNAc, N-acetylglucosamine; HECTD3, homologous to the E6-AP carboxyl terminus 3; HTRA2, high temperature requirement protein A2; IAP, inhib-itor of apoptosis proteins; ICAD, inhibitor of caspase-activated DNase; IKK, IκB kinase; IL-8, interleukin-8; ITCH, E3 ubiquitin-protein ligase Itchy homolog; IκB, inhibitors of the κB protein; JNK, Jun NH2-terminal kinase; LUBAC, linear ubiquitin chain as-sembly complex; MAPK, mitogen-activated protein kinase; MAPKKK, MAP kinase kinase kinase; MKRN1, makorin ring finger protein 1; MOMP, mitochondrial outer membrane permeabilization; MPD, membrane proximal domain; NF-κB, nuclear factor-κB; NO, nitric oxide; Noxa, nutrient-deprivation autophagy factor-1; OGA, O-GlcNAcase; OMI, omi stress-regulated endoprotease; PARP1, poly (ADP–ribose) polymerase 1; PI3K, phospho-inositide 3-kinase; PKC, protein kinase C; PLAD, pre-ligand assembly domain; Plk1, polo-like kinase 1; Plk3, polo-like kinase 3; PTEN, phosphatase and tensin homologue; PTM, posttranslational modification; Puma, P53 upregulated modulator of apoptosis; RAF1, RAF proto-oncogene serine/threonine-protein kinase; RIPK1/RIP1, receptor-interacting serine/threonine-protein kinase 1; ROS, reactive oxygen species; RSK2, ribosomal S6 kinase 2; SFK, Src family kinases; SHP-1, Src homology domain 2 (SH2)-containing tyrosine phosphatase-1; SMAC, second mitochondria-derived activator of caspase; SUMO, Small ubiquitin-related modifier; tBid, truncated Bid; THD, TNF Homology Domain; TM, Transmembrane domain; TNBC, triple negative breast cancer cells; TNF, tumor necrosis factor; TNFR1, tumor necrosis factor receptor 1; TRAF2, TNF receptor associated protein 2; TRAIL, TNF-related apoptosis-inducing ligand; TRIM13, Tripartite Motif-Containing Protein 13; XIAP, X-chromosome-linked inhibitor of apoptosis protein.
